# Factors associated with prolonged ICU stay: a retrospective analysis

**DOI:** 10.1186/cc12625

**Published:** 2013-06-19

**Authors:** FG Zampieri, F Colombari, C de Batista Lovatto Pastore, C Santoro, D Haib, JP Ladeira

**Affiliations:** 1 Hospital Alemão Oswaldo Cruz, Paraíso, São Paulo, SP, Brazil

## Introduction

Critically ill patients frequently stay on the ICU for prolonged periods. Prolonged ICU stay (PIS) is associated with increased costs, resource use and family burden. Nevertheless, risk factors at admission associated with prolonged ICU stay are only partially described. The objective was to evaluate factors associated with prolonged ICU stay on a mixed ICU.

## Methods

Retrospective analysis of 3,257 patients admitted to a tertiary hospital in São Paulo, Brazil. Twenty-seven relevant variables that were clinically associated with prolonged (≥14 days) were included on a univariate analysis. Variables included demographic data, reason for admission, type of admission (clinical, elective surgery, emergency surgery), previous status performance, presence of comorbidities, illness severity (assessed by SAPS 3 score), laboratorial data and need for organ support device (vasopressors, mechanical ventilation, dialysis) on the day of admission. A multivariate analysis was performed to identify variables independently associated with PIS.

## Results

In total, 203 (6.3%) of the 3,257 patients admitted in the analyzed period stayed on the ICU for at least 14 days. Hospital mortality was higher in patients with PIS (49.7% vs. 9.8%; *P <*0.01). On multivariate analysis, SAPS 3 (OR = 1.03, CI = 1.01 to 1.04), reduced status performance (dependency for one or more daily activities - OR = 1.71, CI = 1.18 to 2.46), bedridden status (OR = 1.91, CI = 1.08 to 3.38), emergency surgery (OR = 2.87, CI = 1.27 to 6.51), admission due to intracranial mass effect (OR = 4.46, CI = 1.16 to 17.04), admission from the ward (OR = 3.35, CI = 1.05 to 10.63) and hospital transfer (OR = 5.23, CI = 1.62 to 16.91) were independently associated with PIS. Age was not related to PIS. No comorbidity or organ support device was independently associated with PIS.

## Conclusion

In a large database of critically ill patients, global illness severity, baseline status performance and emergency surgery were related to PIS. No comorbidity or need for organ support device was associated with PIS.

